# Longitudinal profiling of serum ADAM17 across clinical stages in multiple myeloma: a dynamic biomarker and its association with T cell alterations

**DOI:** 10.3389/fmolb.2026.1768193

**Published:** 2026-01-30

**Authors:** Jiao Qu, Yancheng Li, Chenchen Li, Di Wu, Yulin Cao, Shumei Xiao, Xingshuo Bao, Qiubai Li, Zhichao Chen, Lei Chen

**Affiliations:** 1 Department of Hematology, Union Hospital, Tongji Medical College, Huazhong University of Science and Technology, Wuhan, China; 2 Department of Rheumatology and Immunology, Union Hospital, Tongji Medical College, Huazhong University of Science and Technology, Wuhan, China; 3 Institute of Hematology, Jingmen Central Hospital, Jingmen, Hubei, China

**Keywords:** ADAM17, biomarker, CD62L, multiple myeloma, T cells

## Abstract

**Background:**

Multiple myeloma (MM) is the second most common hematologic malignancy in adults. Owing to marked heterogeneity in pathogenesis, clinical presentation and prognosis, the 5-year survival rate remains approximately 50%. Robust and clinically actionable biomarkers are therefore urgently needed to refine risk stratification, guide therapeutic decisions and improve prognostic accuracy. ADAM17 (A Disintegrin and Metalloproteinase-17) plays a pivotal role in inflammation, tissue homeostasis and tumorigenesis. Although the research in MM was limited, the existing evidence suggests that ADAM17 may be involved in the pathological process of the disease.

**Methods:**

In this study, the serum ADAM17 concentrations of 26 MM patients at three different clinical stages (newly diagnosed, remission and progression) were detected by ELISA, and the correlation between ADAM17 concentrations and clinical parameters was analyzed. The expression levels of CD62L on bone marrow T cells of MM patients and healthy donors were compared by flow cytometry. In addition, the above findings were verified in the expanded cohort using the GEO public data set.

**Results:**

Serum ADAM17 concentrations increased progressively from remission to progression and at diagnosis. Levels aligned with DS, ISS and R-ISS staging systems and were strongly associated with renal function. Compared with healthy controls, T cells from MM patients displayed significantly reduced CD62L expression across CD3^+^, CD4^+^ and CD8^+^ subsets, with the most pronounced loss on CD8^+^ T cells. CIBERSORT analysis revealed significantly higher bone-marrow infiltration of CD8^+^ T cells in patients with low *versus* high ADAM17 expression.

**Conclusion:**

Our data identify ADAM17 as an easily quantifiable, longitudinal biomarker that concurrently reflects tumor development stage and renal function damage in MM patients. Incorporation of ADAM17 into existing risk algorithms may enhance prognostic precision and enable earlier, patient-tailored intervention.

## 1 Introduction

Multiple myeloma (MM) represents the second most frequent hematologic malignancy in adults, originating from the clonal expansion of malignant plasma cells within the bone marrow microenvironment. It constitutes approximately 1.3% of all cancer cases and 10%–15% of hematologic malignancies (Yu et al., 2023; Rajkumar, 2024). Over the past decade, the introduction of novel therapies—including proteasome inhibitors, immunomodulators, monoclonal antibodies, autologous stem-cell transplantation, and cellular immunotherapy—has substantially improved treatment outcomes. Nevertheless, due to the considerable heterogeneity in its pathogenesis and clinical presentation, the 5-year survival rate remains approximately 50% (Rees and Kumar, 2024; Avet-Loiseau et al., 2025). Consequently, identifying robust and clinically relevant biomarkers is imperative to refine risk stratification, guide therapeutic decision-making, and enhance prognostic precision.

ADAM17 (A Disintegrin and Metalloproteinase-17), belonging to a multifunctional ADAM protein family, plays a crucial role in converting nearby membrane-anchored cytokine precursors into soluble bioactive mediators (Borrell-Pagès et al., 2003; Lambrecht et al., 2018). It has been reported that ADAM17 can shed 90 membrane-associated substrates in their extracellular environment, thereby playing a pivotal role in inflammation, tissue homeostasis, and cancer development (Duffy et al., 2009; Sun L. et al., 2024; Liu et al., 2025). More recently, its relevance has been extended to oncology. One study demonstrated that elevated circulating ADAM17 levels are observed in colorectal cancer compared to healthy controls (Walkiewicz et al., 2017). Nina Hedemann et al. indicated that the concentration of ADAM17 in both serum and ascites of ovarian cancer patients correlates with FIGO stage and residual tumor burden following primary cytoreductive surgery (Rogmans et al., 2021). Up to now, there have been relatively few studies on the role of ADAM17 in myeloma. But they all demonstrated a pathogenic role for ADAM17 in MM progression. For instance, one study showed that transcript levels of ADAM17 were specifically upregulated in melphalan-resistant cells (Mutlu et al., 2012). Furthermore, CX3CL1, which is cleaved by ADAM17, exhibits increasing expression during MM progression that parallels intramedullary microvascular density (Marchica et al., 2019). Together, these findings support a pathogenic role for ADAM17 in MM progression.

We systematically characterized ADAM17 expression in different disease states of MM patients (newly diagnosed, progression and remission stage), delineated its relationship with clinical disease progression, and evaluated its potential as a dynamic biomarker of tumor burden. This work provides a rational basis for incorporating ADAM17 into disease stratification and targeted therapeutic strategies.

## 2 Materials and methods

### 2.1 Human serum collection and enzyme-linked immunosorbent assay (ELISA)

We collected peripheral blood serum samples from 26 MM patients during consecutive clinical visits to Union Hospital, Tongji Medical College, Huazhong University of Science and Technology from January 2020 to December 2024. Serum collection covered three stages: newly diagnosed, post-treatment remission (VGPR/CR), and disease progression. The determination of disease stage is based on the criteria of the International Myeloma Working Group (IMWG). Clinical data and laboratory test data of patients are collected for statistical analysis. We obtained counterpart samples from five healthy donors at our hospital.

Before treatment, venous blood specimens were drawn and centrifuged at 3,000 rpm for 10 min. The upper serum was collected into EP tubes and stored at −80 °C. The serum concentrations of ADAM17 were quantified using a commercial ELISA kit from RUIXIN BIOTECH, according to the manufacturer’s instructions (catalog number RX104791H).

### 2.2 Collection of bone marrow samples for flow cytometry (FCM) analysis and ADAM17 activity assay

We tested and analyzed the bone marrow of 13 MM patients and 16 non-tumor patients. Detailed demographic characteristics and disease stage of MM patients were provided in Supplementary Table S1. The following antibodies were used in this study: CD45-APC (Biolegend, catalogue number: 368516), CD3-FITC (Biolegend, catalogue number:300306), CD4-PE/CY7 (Biolegend, catalogue number: 344612), CD8-APC (Biolegend, catalogue number:344722), CD62L-PE (Biolegend, catalogue number:304806). Upon receipted of bone marrow samples, added PBS for washing. Centrifuged at 1,300 rpm for 5 min, and aliquoted into tubes at 50 μL/tube. Added Human Transtain FcX (Biolegend, catalogue number: 4422302) and incubated at room temperature (RT) for 5–10 min. Then added the pre-configured antibody panel and incubated in the dark at RT for 20 min. Red blood cells (RBCs) were lysed with BD FACS lysis solution (10 min, 25 °C), followed by centrifugation at 1,500 rpm for 5 min. The supernatant was discarded, and washed twice with PBS. Finally, the cells were gently vortexed in 200 μL of cold PBS for flow cytometry analysis.

ADAM17 activity was measured using SensoLyte® 520 TACE (α - Secretase) Activity Assay Kit (ANASPEC). We collected 3 MM patients and three non-tumor samples for ADAM17 activity detection. We washed the samples by PBS for twice. Next, one million cells were collected, and lysed with assay buffer. ADAM17 enzymatic activity was quantified by continuous measurement of fluorescence intensity in a microplate fluorometer (λex 490 nm and λem 520 nm).

### 2.3 Clinical correlation, staging and immune-infiltration analyses of GEO datasets

For GSE136324 and GSE136337, the preprocessed series matrix expression files and corresponding clinical phenotype tables were downloaded by using the GEOquery package in R software. And only cases with complete expression and clinical data were retained for downstream analyses. All analyses were performed on the log2-normalized, gene-level expression matrices provided by GEO, without additional probe collapsing or re-normalization. For each dataset, routine laboratory indices were extracted from the GEO phenotype tables and merged with log2 expression values of ADAM17 by sample ID. To evaluate continuous associations between ADAM17 and clinical laboratory variables, Spearman’s rank correlation coefficients (ρ) and two-sided *P*-values were calculated available measurements, and visualized using scatter plots with linear trend lines. Disease severity was assessed by International Staging System (ISS) stage, Revised International Staging System (R-ISS) and Revised-2 International Staging System (R2-ISS), and differences in ADAM17 expression across ISS/R-ISS/R2-ISS stages were examined using the Kruskal–Wallis test, with boxplots and jittered points were used for visualization. R-ISS integrates ISS, LDH, and high-risk cytogenetics: del (17p), t (4; 14), and t (14; 16). R2-ISS incorporates ISS, LDH, del (17p), t (4; 14), and 1q21 gain/amplification with weighted scoring. Immune cell infiltration was further characterized using the CIBERSORT algorithm. Immune cell fractions were compared between groups using Wilcoxon rank-sum tests, and *P*-values across cell types were adjusted using the Benjamini–Hochberg false discovery rate (FDR) method (FDR <0.05 considered significant). Immune-infiltration patterns associated with ADAM17 were visualized in R using ggplot2, patchwork and pheatmap.

### 2.4 Ethics statement and clinical characteristics of our clinical samples

This research was approved by the Ethics Committee of Tongji Medical College of Huazhong University of Science and Technology. The diagnosis and treatment responses were assessed according to the International Myeloma Working Group (IMWG) criteria and verified against NCCN guidelines.

### 2.5 Statistical analysis

IBM SPSS 22.0, GraphPad Prism 8.0 and R Statistical Computing Environment version 4.3.1 were utilized for conducting all statistical analyses. One-way ANOVA was used for multiple group comparison. Two-tailed *t*-test and Mann–Whitney U test for two-group comparisons, Wilcoxon matched-pair test for paired data. Spearman’s rank correlation was used for correlation analyses. Significance was set at *P* < 0.05.

## 3 Results

### 3.1 Serum ADAM17 levels are elevated in MM patients compared to healthy donors and correlate with higher-risk disease stages

**FIGURE 1:**
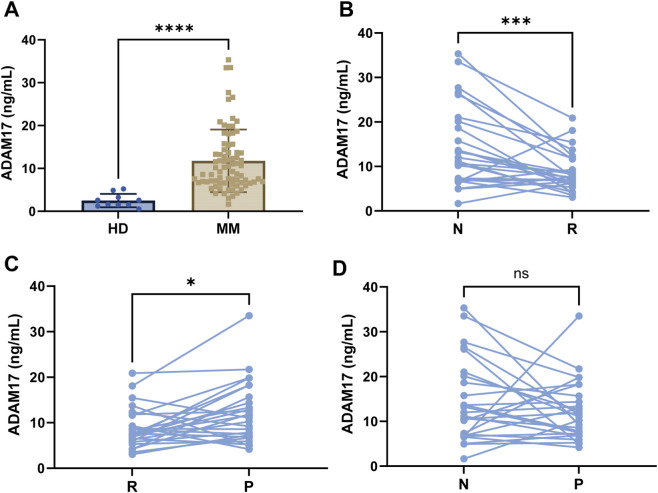
The expression of serum ADAM17 in different risk disease stages of MM patients and healthy donors **(A)** The concentrations of ADAM17 in MM patients (n = 78) and healthy donors (HD) (n = 10). **(B)** Wilcoxon matched-pairs signed rank test compares the difference of ADAM17 expression between newly diagnosed (N) MM patients and remission (R) MM patients (n = 26). **(C)** Wilcoxon matched-pairs signed rank test compares the difference of ADAM17 expression between remission (R) MM patients and MM progression (P) patients (n = 26). **(D)** Wilcoxon matched-pairs signed rank test compares the difference of ADAM17 expression between newly diagnosed (N) MM patients and Progression (P) MM patients (n = 26). Statistically significant differences between each patient group are shown as ***P < 0.001, **P < 0.01, not significant (ns) = *P* > 0.05.

Detailed demographic and disease severity stage of 26 newly diagnosed MM (NDMM) patients are comprehensively documented in Table 1 and disease severity was assessed according to the three risk-stratification systems: Durie-Salmon (DS), ISS, R-ISS. We also collected clinical characteristics of MM patients, including serum albumin (ALB), β2-microglobulin (β2-M), lactate dehydrogenase (LDH) and other routine laboratory indices. Those characteristics were stratified into high and low groups based on the median value of ADAM17 expression, and detail differences were described in Table 2. Then, our results showed that serum ADAM17 levels were significantly higher in MM patients (11.78 ± 7.32 ng/mL) than in healthy adults (2.5 ± 1.55 ng/mL) (Figure 1A). We further compared ADAM17 concentrations across different clinical stages in the 26 patients: at newly diagnosed (14.32 ± 9.19 ng/mL), remission (8.74 ± 4.43 ng/mL), and progression (12.27 ± 6.66 ng/mL). Compared with the remission phase, serum ADAM17 levels were significantly elevated both at newly diagnosed (Figure 1B) and progression phases (Figure 1C). However, no statistically significant difference was observed between the newly diagnosed and progression stages (Figure 1D).

**TABLE 1:** Clinicopathological features of 26 NDMM patients from the Union Hospital, Tongji Medical College.

Characteristic	n
Age (years)	
>60	16
≤60	10
Gender	
Male	14
Female	12
DS stage	
I	5
II	5
III	16
ISS stage	
I	5
II	5
III	16
R-ISS stage	
I	5
II	12
III	9

Abbreviations: DS, Durie-Salmon; ISS, international staging system; R-ISS, revised international staging system.

**TABLE 2:** Clinical characteristics of MM patients with high serum ADAM17 and low serum ADAM17 groups.

Characteristic	ADAM17 ≥ 10 ng/mL	ADAM17 < 10 ng/mL	*t/Z*	*P* value
Number, n, %	39 (50)	39 (50)	-	-
WBC(G/L)	5.43 ± 2.77	4.54 ± 1.64	1.122	0.289
Hb (g/L)	97.36 ± 25.27	100.97 ± 33.08	1.112	0.292
PLT (G/L)	190.54 ± 83.45	144.79 ± 65.71	6.318	0.012*
Alb (g/L)	37.02 ± 5.73	38.26 ± 7.68	0.462	0.497
Cr (μmol/L)	112.5 ± 73.29	102.73 ± 82.66	2.494	0.114
UA (μmol/L)	375.75 ± 140.16	322.16 ± 153.13	3.553	0.059
LDH (U/L)	300.53 ± 201.12	208.62 ± 116.12	9.693	0.002*
eGFR (ml/(min/1.73m^2^))	82.39 ± 94.6	79.16 ± 32.12	3.006	0.083
Calcium (mmol/L)	2.28 ± 0.29	2.28 ± 0.37	0.351	0.553
β2-M(mg/L)	6.87 ± 4.28	4.73 ± 3.51	6.860	0.009*

Abbreviations: WBC, white blood cell; Hb, hemoglobin; PLT, platelets; Alb, albumin; Cr, creatinine; UA, uric acid; LDH, lactate dehydrogenase; eGFR, estimated glomerular filtration rate; β2-M, β2-microglobulin.

### 3.2 Serum ADAM17 levels correlate with clinical risk stratification

**FIGURE 2:**
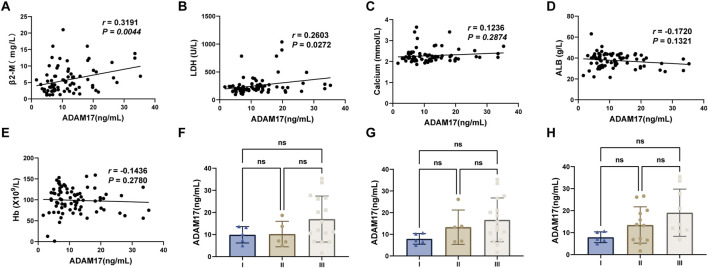
The relationship between serum ADAM17 levels and other clinical parameters The correlation between serum ADAM17 levels and **(A)** β2-M, **(B)** LDH, **(C)** calcium, **(D)** ALB and **(E)** Hb. Serum ADAM17 levels expression among different **(F)** DS stages, **(G)** ISS and **(H)** R-ISS stages. Not significance (ns) = *P* > 0.05.

To investigate whether ADAM17 expression levels can serve as an indicator of disease progression, we performed a correlation analysis between ADAM17 and clinical parameters in MM patients. The results revealed significant associations between ADAM17 and parameters of the DS, ISS and R-ISS staging systems, demonstrating positive correlations with β2-M (Figure 2A), LDH (Figure 2B), and calcium (Figure 2C), and inverse correlations with ALB (Figure 2D) and hemoglobin (Hb) (Figure 2E). Clinical characteristics of MM patients with high serum ADAM17 and low serum ADAM17 groups have been detailed description in Table 2. Further analysis of 26 NDMM patient serum revealed that ADAM17 levels exhibited alignment with DS (Figure 2F), ISS (Figure 2G) and R-ISS (Figure 2H) staging. The serum ADAM17 levels in relation to stage and risk stratification was described in detail in Table 3. ADAM17 levels showed an incremental increase with advancing stage in DS, ISS and R-ISS staging systems. However, the differences did not reach statistical significance, a finding that is likely attributable to the limited sample size.

**TABLE 3:** The serum ADAM17 levels in relation to stage and risk stratification.

Tested parameters	Patient groups	Number of patients (n)	ADAM17 level: Mean ± SD (ng/mL)	*P* value
DS	Stage I	5	9.87 ± 3.73	0.286
	Stage II	5	10.21 ± 5.77	
	Stage III	16	17 ± 10.41	
ISS	Stage I	5	7.88 ± 2.46	0.102
	Stage II	5	13.29 ± 7.93	
	Stage III	16	16.66 ± 10.12	
R-ISS	Stage I	5	7.88 ± 2.45	0.059
	Stage II	12	13.46 ± 8.25	
	Stage III	9	19.06 ± 10.72	

Abbreviations: DS, Durie-Salmon; ISS, international staging system; R-ISS, revised international staging system.

### 3.3 Higher serum ADAM17 levels indicated poor renal function in MM patients

**FIGURE 3:**
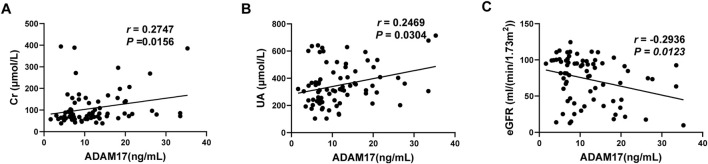
The relationship between serum ADAM17 levels and renal function markers The correlation between serum ADAM17 levels and **(A)** Uric Acid (UA), **(B)** estimated glomerular filtration rate (eGFR), **(C)** Creatinine (Cr).

To further investigate the potential of serum ADAM17 in characterizing disease status in MM patients, we demonstrated its precise reflection of renal function, showing significant positive correlations with creatinine (Cr) and uric acid (UA) levels, and a negative correlation with the estimated glomerular filtration rate (eGFR) (Figures 3A–C).

### 3.4 ADAM17 levels correlate with clinical stage risk stratification in GEO datasets

**FIGURE 4:**
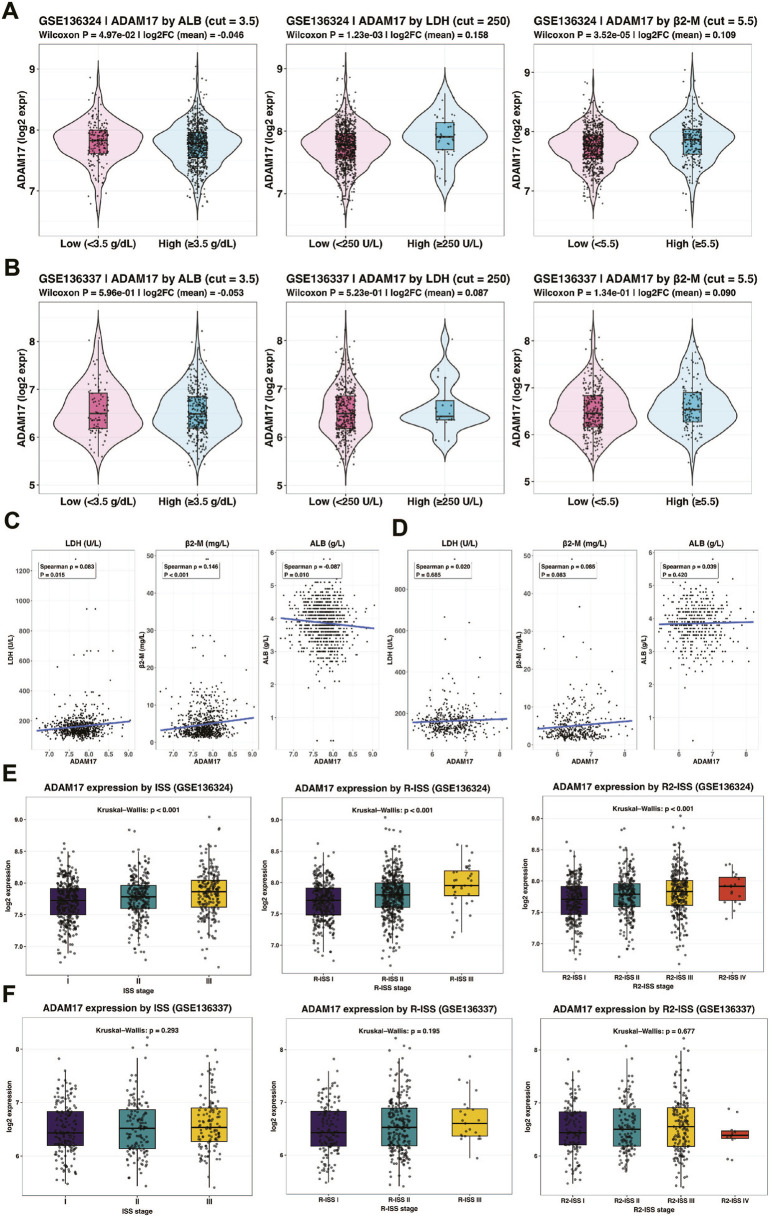
ADAM17 expression across clinical subgroups, clinical risk stages, and laboratory correlations in public GSE136324 and GSE136337 datasets. Violin plots showing ADAM17 log2 expression in **(A)** GSE136324 and **(B)** GSE136337 stratified by key clinical indicators. ALB was dichotomized at 3.5 g/dL, β2-M at 5.5 mg/L, and LDH at 250 U/L. Group differences were assessed using Wilcoxon rank-sum tests, with mean log2 expression differences indicated. Scatter plots illustrating the correlations between continuous ADAM17 expression and clinical laboratory variables—including LDH (U/L), β2-M (mg/L), and ALB (g/dL)—in **(C)** GSE136324 and **(D)** GSE136337. Spearman correlation coefficients (ρ) and two-sided *P* values are shown, with linear trend lines overlaid. Boxplots from left to right showing log2 ADAM17 expression in **(E)** GSE136324 and **(F)** GSE136337 according to ISS, R-ISS, and R2-ISS staging, respectively.

To further validate the above findings with an expanded sample size, we analyzed publicly available datasets. In the GSE136324 cohort, patients were stratified based on clinical parameters using thresholds of ALB <3.5 g/dL, β2-M> 5.5 mg/L, and LDH >250 U/L. We observed significantly higher ADAM17 expression in patients with ALB <3.5 g/dL, β2-M > 5.5 mg/L, and LDH >250 U/L. (Figure 4A). Consistent results were obtained in GSE136337 validation dataset (Figure 4B). Furthermore, ADAM17 expression showed significant positive correlations with LDH and β2-M, and a negative correlation with ALB (Figure 4C) and it was also validated in GSE136337(Figure 4D). We also downloaded the ISS staging risk stratification from the clinical patient information in the GEO dataset. When patients were categorized according to ISS stages, ADAM17 expression demonstrated a progressive increase with advancing disease stage (Figure 4E). It is worth noting that, based on the cytogenetic results provided in the clinical metadata, we re-stratified the patients in the dataset according to the R-ISS and R2-ISS risk criteria. The result was consistent with the above, that ADAM17 expression demonstrated a progressive increase with advancing disease stage (Figure 4E). A similar trend was also observed in GSE136337 dataset (Figure 4F). Collectively, these findings corroborate our clinical serological data, confirming that ADAM17 reliably reflects disease status in MM patients at both the transcriptional and protein levels.

### 3.5 Healthy donors had higher CD62L expression on T cell than MM patients

**FIGURE 5:**
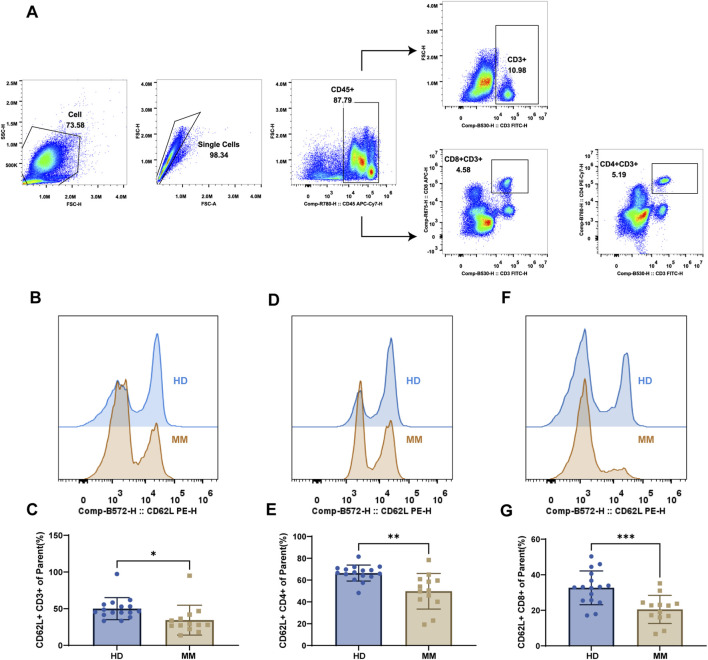
Detection of CD62L expression on the T cell in MM bone marrow **(A)** The gating strategy for flow cytometry experiment. In brief, we first gated on the total cell population, excluded doublets, and identified CD45 positive cells. Subsequently, we gated T cell subsets based on characteristic markers: CD3^+^ T cells (CD3), CD4^+^ T cells (CD3, CD4), and CD8^+^ T cells (CD3, CD8). Finally, we assessed changes in CD62L expression in these distinct cell populations. **(B)** Representative histogram of CD62L fluorescence and **(C)** quantification of CD62L positive CD3^+^ T cell. **(D)** Representative histogram of CD62L fluorescence and **(E)** quantification of CD62L positive CD4^+^ T cell. **(F)** Representative histogram of CD62L fluorescence and **(G)** quantification of CD62L positive CD8^+^ T cell. Statistically significant differences between each patient group are shown as *P < 0.05, **P < 0.01, ***P < 0.001.

To elucidate the physiological role of ADAM17 in MM bone marrow, we measured ADAM17 activity in bone marrow from healthy controls and MM patients, and found it was significantly elevated in MM (Supplementary Figure S1). Next, we focused on its function as a pivotal nexus linking inflammation and cancer. Given the critical contribution of CD8^+^ T cell mediated immunity in controlling MM within the bone marrow microenvironment, and considering that ADAM17 directly modulates T cell phenotype through CD62L shedding, we collected bone marrow samples from both healthy donors and MM patients to examine changes in CD62L expression on T cells. This approach allows simultaneous assessment of ADAM17 activity and T cell phenotypic and functional alterations in the marrow niche. Firstly, we present the flow cytometric gating strategy (Figure 5A). Then, compared to the healthy donors, T cells from MM patients exhibited significantly reduced CD62L expression across CD3^+^ (Figures 5B,C), CD4^+^ (Figures 5D,E), and CD8^+^ T (Figures 5F,G) cell subsets, with a particularly pronounced decrease observed on CD8^+^ T cells.

### 3.6 Estimation CD8^+^ T cell infiltration of MM patients with low/high ADAM17 expression

**FIGURE 6:**
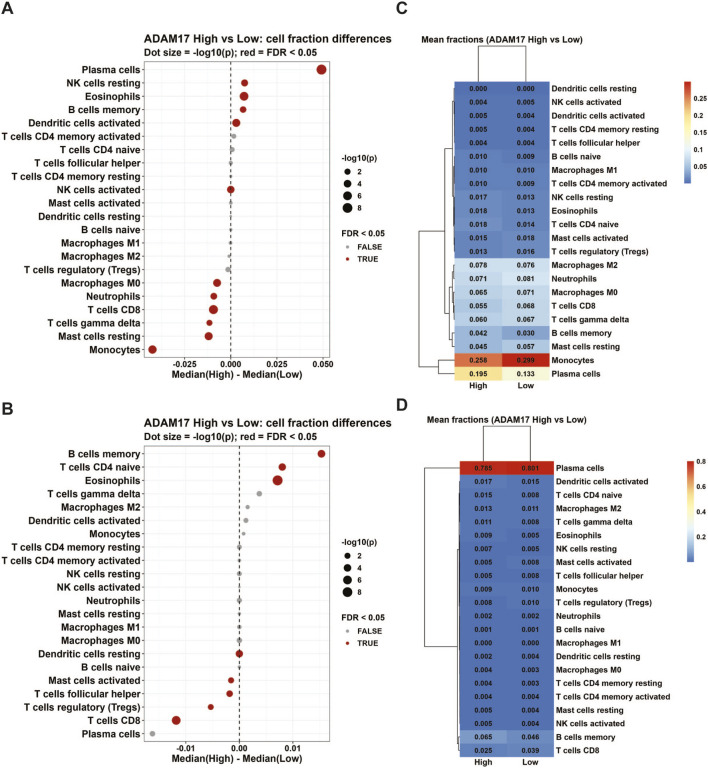
ADAM17 expression in public GSE136324 and GSE136337 datasets. Immune cell fractions were estimated from bulk tumor expression using CIBERSORT with the LM22 signature after back-transforming the GEO log2 expression values (2^expression). Samples with CIBERSORT *P* < 0.05 were retained. Patients were divided into ADAM17-high and ADAM17-low groups by the cohort-specific median. Dot plots show differences in estimated cell fractions between ADAM17-high and ADAM17-low groups (High *versus* Low) in **(A)** GSE136324 and **(B)** GSE136337 datasets; dot size reflects–log10(*P*) from Wilcoxon rank-sum tests, and red dots indicate FDR <0.05 (Benjamini–Hochberg). Heatmaps display mean immune cell fractions in ADAM17-high *versus* ADAM17-low groups in **(C)** GSE136324 and **(D)** GSE136337 datasets.

We further employed the CIBERSORT algorithm to analyze GEO bulk RNAseq data for evaluating CD8^+^ T cell infiltration in MM bone marrow. The results demonstrated that patients with low ADAM17 expression exhibited a significant increase in bone marrow CD8^+^ T cell infiltration compared to those with high ADAM17 expression, it was confirmed in both GSE136324 (Figures 6A,C) and GSE136337 (Figures 6B,D) datasets.

## 4 Discussion

The pathogenesis of multiple myeloma (MM) is characterized by marked biological and clinical heterogeneity, and a substantial proportion of patients eventually develop relapse or progressive disease (Fandrei et al., 2025; Kündgen et al., 2025). Although the International Staging System (ISS) is widely used for risk stratification, its discriminatory power remains limited in guiding individualized treatment strategies or reliably predicting patient-specific outcomes (Sun C. et al., 2024; Yan et al., 2025). There is therefore a pressing need for novel biomarkers—used either independently or in combination with the ISS or R-ISS framework—to enhance prognostic accuracy, steer precision medicine, alleviate disease-related symptoms, and improve quality of life.

ADAM17 orchestrates the ectodomain shedding of multiple cytokines and cognate receptors, including tumor necrosis factor-α (TNF-α), TNF receptors 1/2, interleukin-6 (IL-6) and the IL-6 receptor. Dysregulated ADAM17 activity has been documented across several solid tumors, where it fuels proliferation, angiogenesis and immune evasion (Düsterhöft et al., 2019; Xie et al., 2024). However, its quantitative relationship with MM disease activity remains unexplored. Here we interrogated circulating ADAM17 as a dynamic indicator of tumor burden and investigated its clinical utility in MM.

We first found that serum ADAM17 was markedly elevated in MM patients relative to healthy donors. To eliminate inter-individual genetic noise, we longitudinally sampled the same patients at three clinically annotated time-points: newly diagnosed, confirmed remission and progression stages. Pairwise within-subject comparison revealed a progressive increase in ADAM17 concentration from remission to progression and diagnosis, demonstrating that the protease tracks faithfully with disease activity. Further correlation analysis revealed that ADAM17 levels demonstrated significant positive correlations with β2-M, LDH, and calcium, while showing a marked inverse correlation with ALB, suggesting a potential link to DS, ISS and R-ISS staging. Specifically, ADAM17 concentrations demonstrated an increasing trend corresponding with advancing disease stage. The analysis of 26 NDMM patient serums also revealed that ADAM17 levels exhibited alignment with DS, ISS and R-ISS staging. However, this trend did not reach statistical significance, likely due to the limited sample size and substantial interpatient heterogeneity. Notably, we also observed a strong association between ADAM17 expression and renal function: it correlated positively with creatinine and negatively with estimated glomerular filtration rate (eGFR). These findings are consistent with previous reports indicating that renal ADAM17 is upregulated in both acute and chronic kidney injury, where it promotes the activation of TNF-α and EGFR ligands, leading to myeloma-related renal damage (Mulder et al., 2012; Palau et al., 2019). To further validate the above clinical findings, we expanded our analysis to public datasets and confirmed that ADAM17 expression—both at the transcriptional level and in serum—consistently reflects disease status in MM patients, corroborating our initial serological observations.

The quantity and functional competence of CD8^+^ T cells in the tumor microenvironment are critical determinants of anti-tumor immune efficacy (Davis et al., 2024). Interestingly, high ADAM17 expression has frequently been reported to suppress CD8^+^ T cell function. One study revealed that ADAM10/ADAM17 mediate the cleavage of PD-L1 from the surface of tumor cells, resulting in CD8^+^ T cell apoptosis and diminished anti-tumor immunity (Orme et al., 2020). Additionally, another recent study demonstrated that inhibition of ADAM17 enhances the responsiveness of both murine and human CD8^+^ T cells to stimulation by IL-2, IL-15, and IFN-γ (Sun L. et al., 2024). Given that ADAM17 directly modulates T cell phenotype through CD62L shedding, we focused on this mechanism (Scheller et al., 2011). From a functional perspective, reduced CD62L expression on circulating T cells may impair the recirculation of leukemia antigen-specific T cells to secondary lymphoid organs (Wedepohl et al., 2012), where they are typically activated by antigen-presenting cells. Thus, low CD62L expression on T cells could contribute to inadequate anti-myeloma immune surveillance by disrupting CD62L-dependent T cell trafficking and priming against myeloma-associated antigens (Sopper et al., 2017).

We collected bone marrow samples from both MM patients and healthy donors, and analyzed T cell phenotypic changes. Compared to healthy donors, T cells from MM patients exhibited significantly reduced CD62L expression across CD3^+^, CD4^+^, and CD8^+^ T cell subsets, with a particularly pronounced decrease observed on CD8^+^ T cells. We further employed the CIBERSORT algorithm to analyze sequencing data for evaluating immune cell infiltration. The results demonstrated that patients with low ADAM17 expression exhibited a significant increase in bone marrow CD8^+^ T cell infiltration compared to those with high ADAM17 expression. This finding corroborates our previous clinical phenotyping data and is consistent with existing literature reports. Based on these observations, we hypothesize that increased ADAM17 activity in the MM bone marrow microenvironment promotes CD62L shedding from T cells, leading to partial impairment of T cell function and consequently compromised tumor cell surveillance.

Collectively, our results identify ADAM17 as a readily quantifiable, longitudinal biomarker that simultaneously reflects tumor burden, inflammatory tone and renal compromise in MM. Integration of ADAM17 into existing risk algorithms may enhance prognostic resolution and facilitate early, patient-tailoured intervention. This study has several limitations that should be addressed in future investigations. First, the patient cohort is relatively small and from a single center, incorporating multi-center data would strengthen the robustness of our conclusions. Additionally, ADAM17 can nearly shed 90 membrane-associated substrates from diverse cell types to facilitate intercellular communication and interpretation of the extracellular environment. Our future work will systematically investigate the relationship between ADAM17 and additional shedding substrates to elucidate its broader pathogenic mechanisms in MM, thereby advancing the development of ADAM17 as a therapeutic target for this disease.

## Data Availability

The original contributions presented in the study are included in the article/Supplementary Material, further inquiries can be directed to the corresponding author.

## Supplementary material

SUPPLEMENTARY FIGURE S1 Detection of ADAM17 activity between the bone marrow of MM patients and non-tumor samples. ADAM17 activity was detected in MM patients (n = 3) and healthy donors (HD) (n = 3).
