# Supplementary material for: Longitudinal profiling of serum ADAM17 across clinical stages in multiple myeloma: a dynamic biomarker and its association with T cell alterations
Source: Front Mol Biosci. 2026 Jan 30;13:1768193. doi: 10.3389/fmolb.2026.1768193 (PMC12900718; doi:10.3389/fmolb.2026.1768193)
Supplement: Supplementary file 1 [file Table1.docx]

**Supplementary Table 1**

**Clinicopathological features of 13 MM patients from the Union Hospital, Tongji Medical College.**

| Characteristic | n |
| --- | --- |
| Age（years） |  |
| ＞60 | 8 |
| ≤60 | 5 |
| Gender |  |
| male | 3 |
| female | 10 |
| DS stage |  |
| I | 4 |
| II | 1 |
| III | 8 |
| ISS stage |  |
| I | 4 |
| II | 4 |
| III | 5 |
| R-ISS stage |  |
| I | 3 |
| II | 5 |
| III | 5 |

**Abbreviations**: DS, Durie-Salmon; ISS, International Staging System; R-ISS, Revised International Staging System
