# Supplementary figures and images for: Longitudinal profiling of serum ADAM17 across clinical stages in multiple myeloma: a dynamic biomarker and its association with T cell alterations
Source: Front Mol Biosci. 2026 Jan 30;13:1768193. doi: 10.3389/fmolb.2026.1768193 (PMC12900718; doi:10.3389/fmolb.2026.1768193)

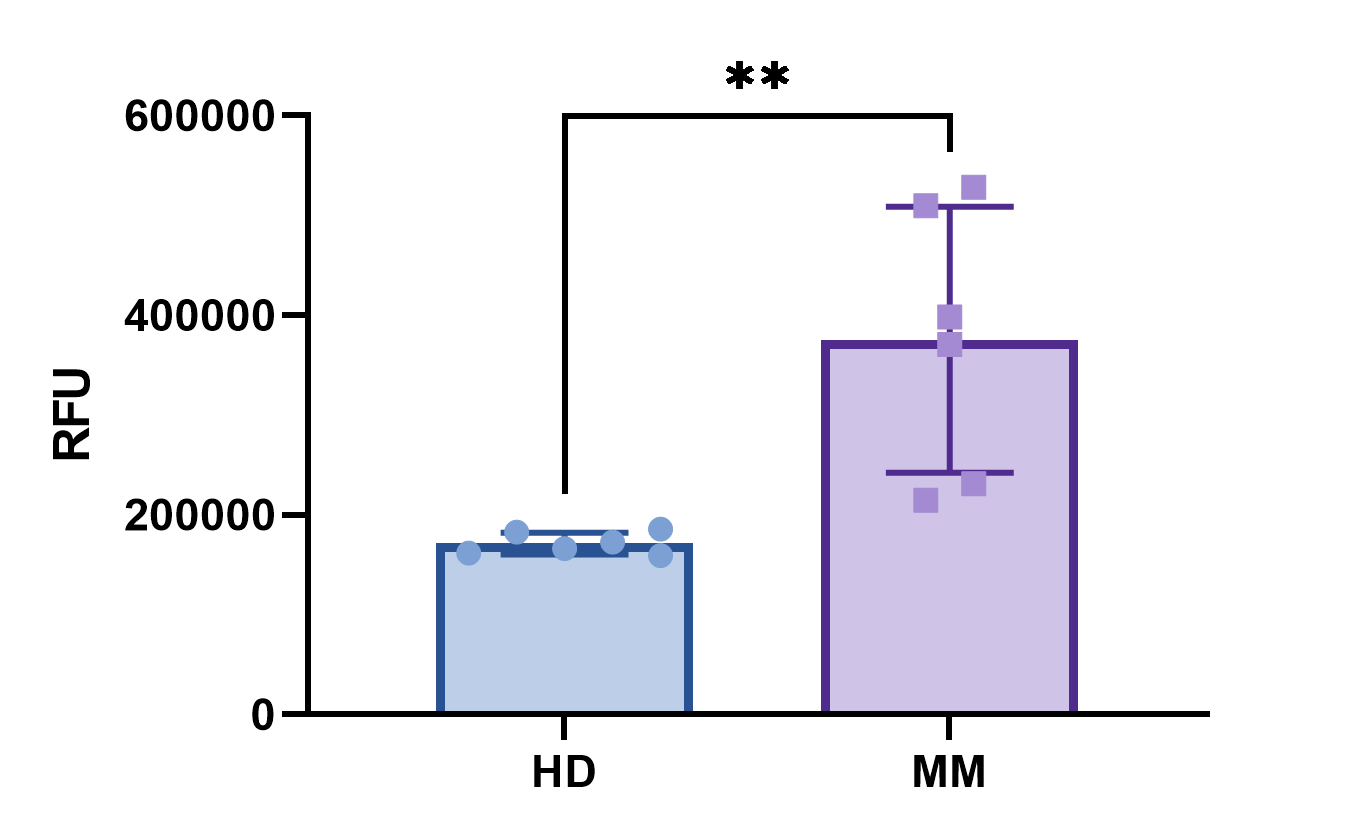

Supplement: Supplementary file 2 [file Image1.tif]
